# SIRT6 enhances telomerase activity to protect against DNA damage and senescence in hypertrophic ligamentum flavum cells from lumbar spinal stenosis patients

**DOI:** 10.18632/aging.202536

**Published:** 2021-02-10

**Authors:** Jianwei Chen, Zude Liu, Hantao Wang, Lie Qian, Zhanchun Li, Qingxin Song, Guibin Zhong

**Affiliations:** 1 Department of Spine Surgery, Department of Orthopedics, Renji Hospital, School of Medicine, Shanghai Jiao Tong University, Shanghai, China; 2 Medical Department, Baoshan Branch of Renji Hospital, School of Medicine, Shanghai Jiao Tong University, Shanghai, China

**Keywords:** SIRT6, senescence, DNA damage, telomere dysfunction, telomerase activity

## Abstract

Lumbar spinal stenosis (LSS) is a condition wherein patients exhibit age-related fibrosis, elastin-to-collagen ratio reductions, and ligamentum flavum hypertrophy. This study was designed to assess the relationship between SIRT6 and telomerase activity in hypertrophic ligamentum flavum (LFH) cells from LSS patients. We observed significant reductions in SIRT6, TPP1, and POT1 protein levels as well as increases in telomerase reverse transcriptase (TERT) levels and telomerase activity in LFH tissues relative to non- hypertrophic ligamentum flavum (LFN) tissues. When SIRT6 was overexpressed in these LFH cells, this was associated with significant increases in telomerase activity and a significant reduction in fibrosis-related protein expression. These effects were reversed, however, when telomerase activity was inactivated by hTERT knockdown in these same cells. SIRT6 overexpression was further found to reduce the frequency of senescence-associated β-galactosidase (SA-β-Gal)-positive LFH cells and to decrease p16, MMP3, and L1 mRNA levels and telomere dysfunction-induced foci (TIFs) in LFH cells. In contrast, hTERT knockdown-induced telomerase inactivation eliminated these SIRT6-dependent effects. Overall, our results indicate that SIRT6 functions as a key protective factor that prevents cellular senescence and telomere dysfunction in ligamentum flavum cells, with this effect being at least partially attributable to SIRT6-dependent telomerase activation.

## INTRODUCTION

Lumbar spinal stenosis (LSS) is a relatively common cause of lower back pain in older adults, and it is characterized by age-associated fibrosis, reduced elastin-to-collagen ratios, and ligamentum flavum (LF) hypertrophy [1, 2]. Under normal conditions, the LF is highly elastic and is composed of approximately 80% elastin fibers and 20% collagen fibers, with the relative frequency of collagen fibers in the LF rising as a function of age, degeneration, and tissue hypertrophy [3, 4]. The onset of LSS is associated with a range of different pathological changes including increases in the expression of inflammatory cytokines, matrix metalloproteinases, and pro-fibrotic growth factors [5]. Cellular senescence is thought to be an important driver of intervertebral disc degeneration [6–8]. The specific molecular mechanisms governing LF cell senescence in the context of LSS development, however, remain to be defined.

Sirtuins are NAD+ dependent histone deacetylases that control genomic stabilization, inhibit inflammation, and slow the aging process [9]. Seven total sirtuins (SIRT1-7) have been described in mammals, with each member of this family having different localization patterns and target proteins within cells [10]. SIRT6 is a sirtuin with a high degree of substrate-specificity that plays important roles in maintaining chromosomal integrity and functionality through influencing DNA repair, gene expression, and genomic stabilization [11]. SIRT6 deficiencies have been linked to premature aging phenotypes in mice [12], whereas SIRT6 overexpression is associated with an increased lifespan in these same animals [13], thus suggesting that SIRT6 is a key regulator of both physiological aging and aging-associated disease.

Cellular senescence is closely linked to the structure and function of telomeres, which are located on chromosomal ends and which grow shorter with each round of cell division. When these telomeres reach a critical length, cells cease to divide and are considered to be senescent [14]. Telomeres can be lengthened by telomerase, which is a ribonucleoprotein complex composed of RNA and the human telomerase reverse transcriptase (hTERT) enzyme, and which functions by adding repeating telomere sequences to the 3’ ends of DNA, thereby forestalling senescence and extending the cellular lifespan [15]. Nagai et al. [16] demonstrated that when SIRT6 was knocked down in human chondrocytes, this was associated with premature senescence, telomere dysfunction, and DNA damage. Whether SIRT6 impacts telomerase activity in hypertrophic LF (LFH) cells from LSS patients, however, remains to be determined. The present study was therefore designed to assess how SIRT6 impacts DNA damage, telomere dysfunction, and cellular senescence in these cells, with our hypothesis being that SIRT6 can promote telomerase activation and thereby protect LF cells from aging-related damage.

## RESULTS

### Ligamentum flavum hypertrophy is associated with the downregulation of SIRT6 and the activation of telomerase activity

**Figure 1:**
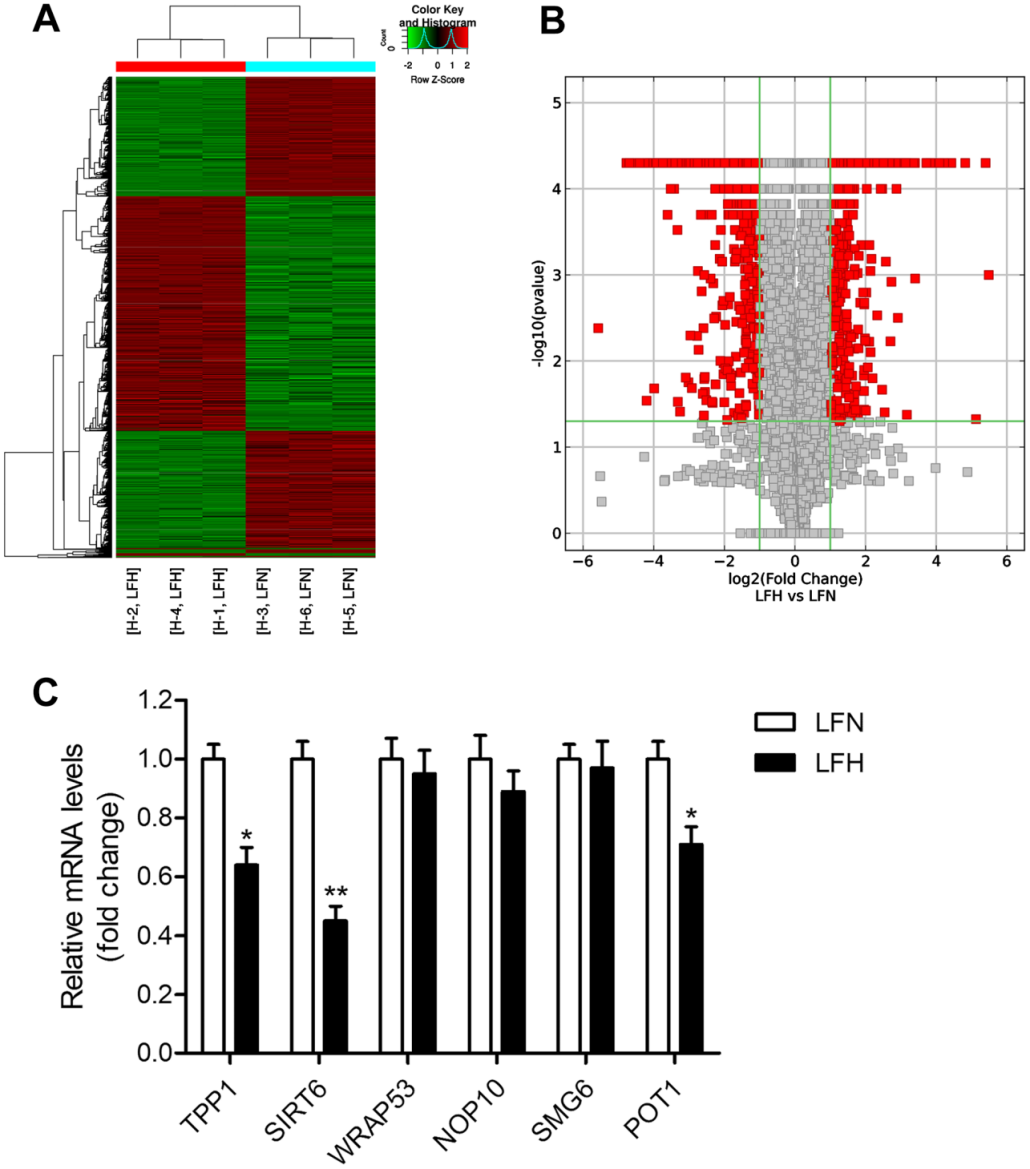
**Detection of differentially expressed genes in hypertrophic ligamentum flavum tissue samples from lumbar spinal stenosis patients.** (**A**) A heat map was used to display differentially expressed genes (DEGs) identified when comparing non-hypertrophic and hypertrophic ligamentum flavum (LFN and LFH, respectively) tissue samples. Up- and down-regulated genes are shown in red and blue, respectively. n = 3. (**B**) DEGs were arranged in Volcano plots. (**C**) Differential SIRT6 and telomere function-related gene expression was confirmed via qRT-PCR, with GAPDH being used as a normalization control. n = 30. *p<0.05, **p<0.01 vs. LFN.

**Figure 2:**
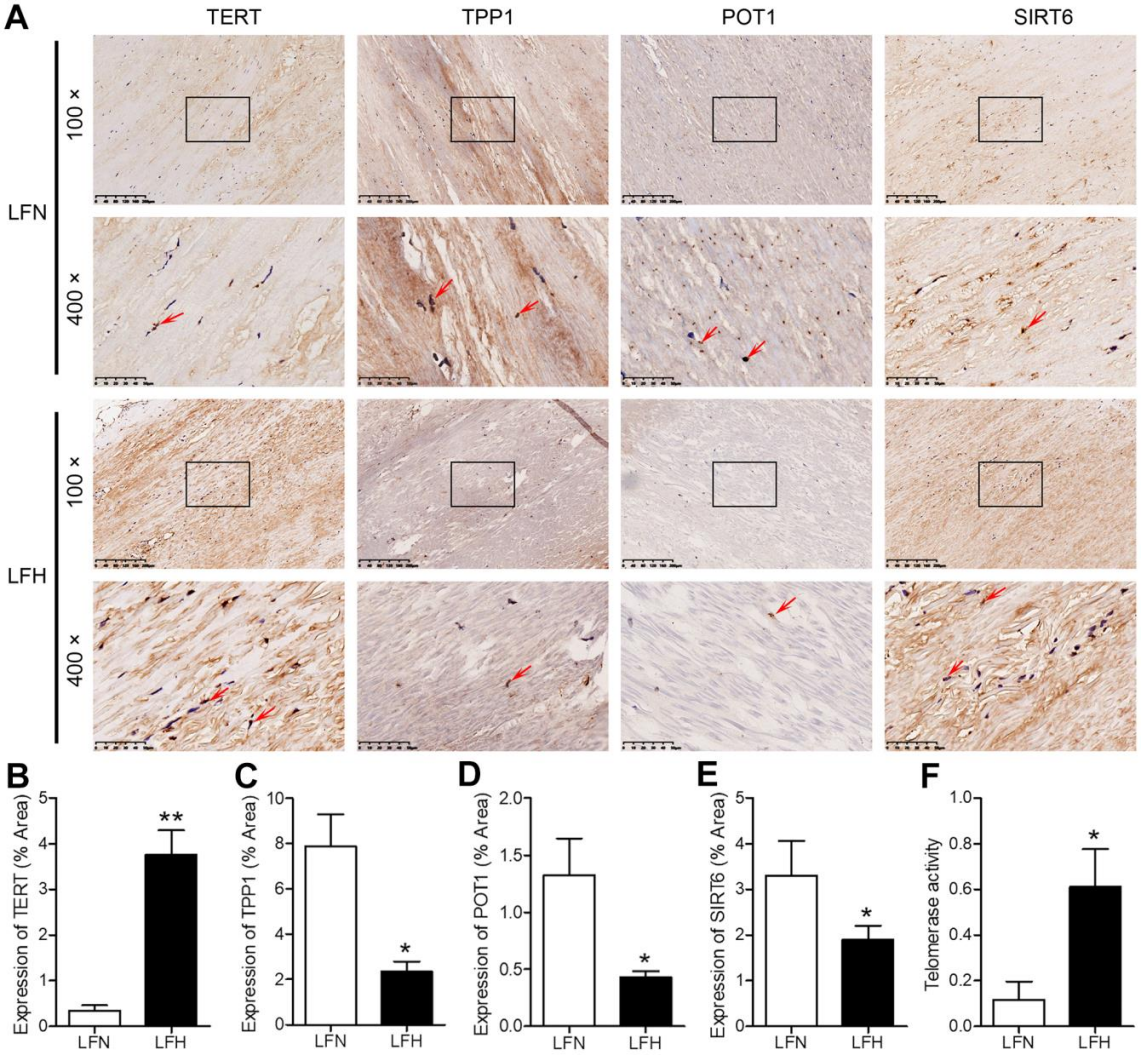
**Analysis of SIRT6 and telomerase activity in human ligamentum flavum samples.** (**A**) TERT, TPP1, POT1, and SIRT6 levels in non-hypertrophic and hypertrophic ligamentum flavum (LFH and LFN, respectively) tissue samples were assessed via immunohistochemistry (IHC). Scale bar = 200 μm or 50 μm. (**B**–**E**) Quantitative analysis of the percent area positive TERT (**B**), TPP1 (**C**), POT1 (**D**) and SIRT6 (**E**) staining area. (**F**) Telomerase activity in LFH and LFN tissue samples as represented by telomerase signal (optical density). n = 30. *p<0.05, **p<0.01 vs. LFN.

When we compared gene expression levels in hypertrophic ligamentum flavum (LFH) and non-hypertrophic ligamentum flavum (LFN) tissue samples from LSS patients, we found that the expression of SIRT6 and of the telomere-related genes POT1 and TPP1 was significantly reduced in LFH samples relative to LFN samples through both transcriptomic high-throughput sequencing and qRT-PCR analyses (Figure 1 and Table 1). In total, 3,935 differentially expressed genes (DEGs) were identified through hierarchical clustering analyses (Figure 1A, 1B and Supplementary Table 1), with 1,952 and 1,983 of these genes being up- and down-regulated, respectively, in LFH tissues. DEGs associated with telomere function are shown in Table 1. Six of these DEGs were selected for qRT-PCR-based validation, revealing TPP1, SIRT6, and POT1 to have been significantly downregulated in LFH samples relative to LFN samples (Figure 1C). This was further supported by immunohistochemical staining analyses, which demonstrated that TERT was upregulated and POT1, TPP1, and SIRT6 was downregulated in LFH tissues at the protein level relative to LFN tissues (Figure 2A–2E). We similarly detected significantly increased telomerase activity in LFH samples relative to LFN samples using a telomerase PCR ELISA kit (Figure 2F). Together, these findings suggested that alterations in the expression of SIRT6 and telomerase activity were associated with LF hypertrophic pathology.

**Table 1:** Differentially expressed genes involved in telomere function.

**Gene Symbol**	**Description**	**Fold change**	**Gene ID**
TPP1	adrenocortical dysplasia homolog	-4.60689	65057
SIRT6	sirtuin 6	-3.16075	51548
WRAP53	WD repeat containing, antisense to TP53	-2.32817	55135
NOP10	NOP10 ribonucleoprotein	-2.04305	55505
SMG6	SMG6 nonsense mediated mRNA decay factor	-2.02718	23293
POT1	protection of telomeres 1	-1.95619	25913

### SIRT6 overexpression enhances telomerase activity and inhibits fibrosis in ligamentum flavum cells

**Figure 3:**
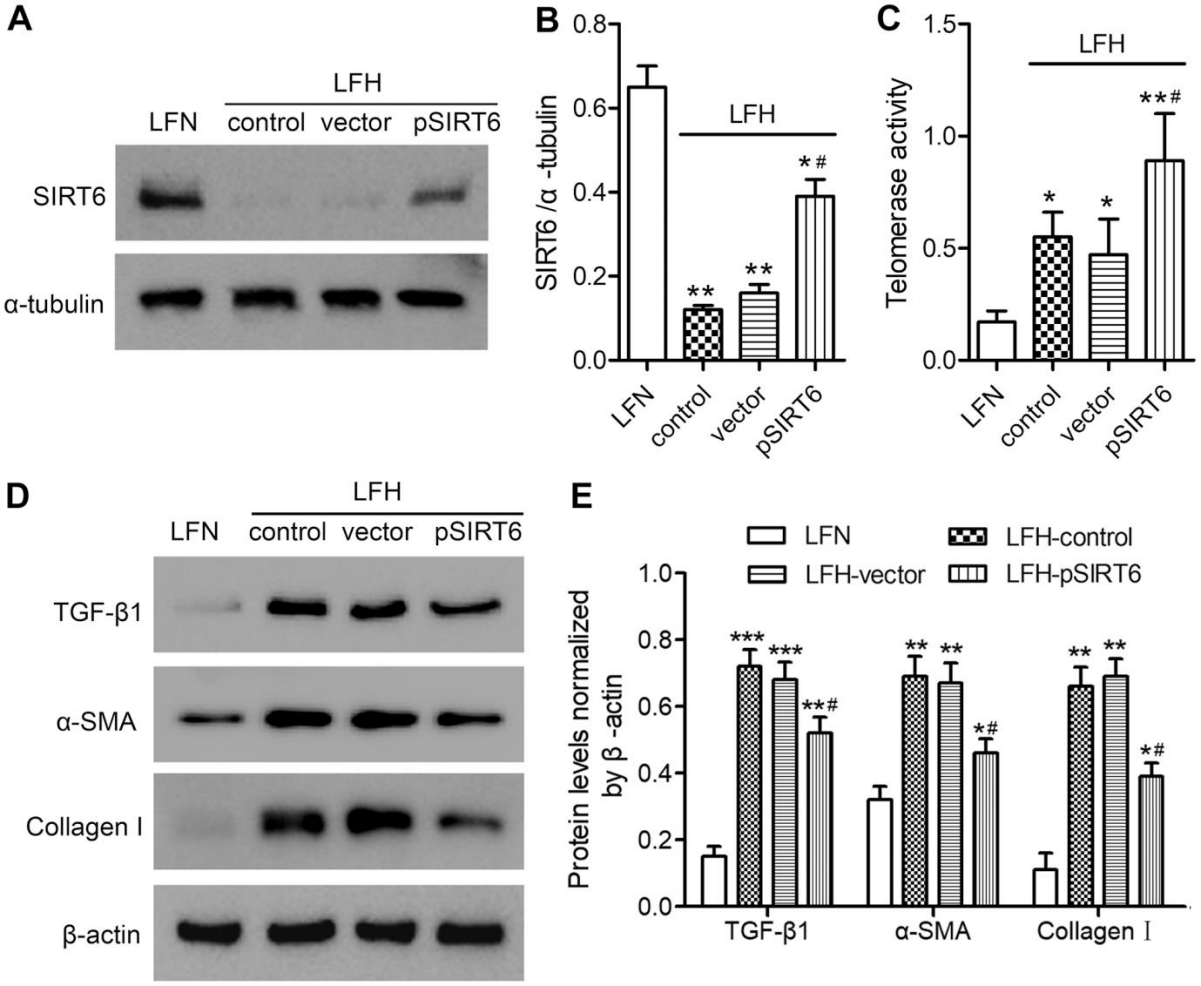
**SIRT6 overexpression enhances telomerase activity and inhibits fibrosis in ligamentum flavum cells.** SIRT6 or control lentiviral vectors were used to transduce LFH cells for 48 h, after which western blotting was used to assess SIRT6 levels in these cells (**A**, **B**). α-tubulin was used for normalization. Uninfected LFH cells and LFN cells were included as controls. (**C**) Telomerase activity in ligamentum flavum cells transduced as indicated was assessed based upon optical density. (**D**, **E**) TGF-β1, α-SMA, and collagen I protein levels were analyzed by western blotting. β-actin was used as a loading control. Data are means ± SD of three replicates. *p<0.05, **p<0.01 vs. LFN. #*p*<0.05 vs. LFH control.

We next assessed the impact of SIRT6 on LF cell functionality by transducing LFH cells for 48 h with a lentivirus encoding human SIRT6, resulting in significant protein-level SIRT6 upregulation relative to untransfected LFH control cells (Figure 3A, 3B and Supplementary Figure 1). This SIRT6 overexpression was associated with significantly enhanced telomerase activity, although this activity remained at a low level in LFN cells (Figure 3C). SIRT6 overexpression was also associated with significant reductions in TGF-β1, α-SMA, and collagen I protein levels in LFH cells (Figure 3D, 3E and Supplementary Figure 2). Together, these findings suggest that SIRT6 can suppress the myofibroblastic differentiation of LFH cells by enhancing telomerase activation.

### SIRT6 overexpression enhances telomerase activity and fibrosis-related protein expression through a mechanism dependent upon hTERT upregulation

**Figure 4:**
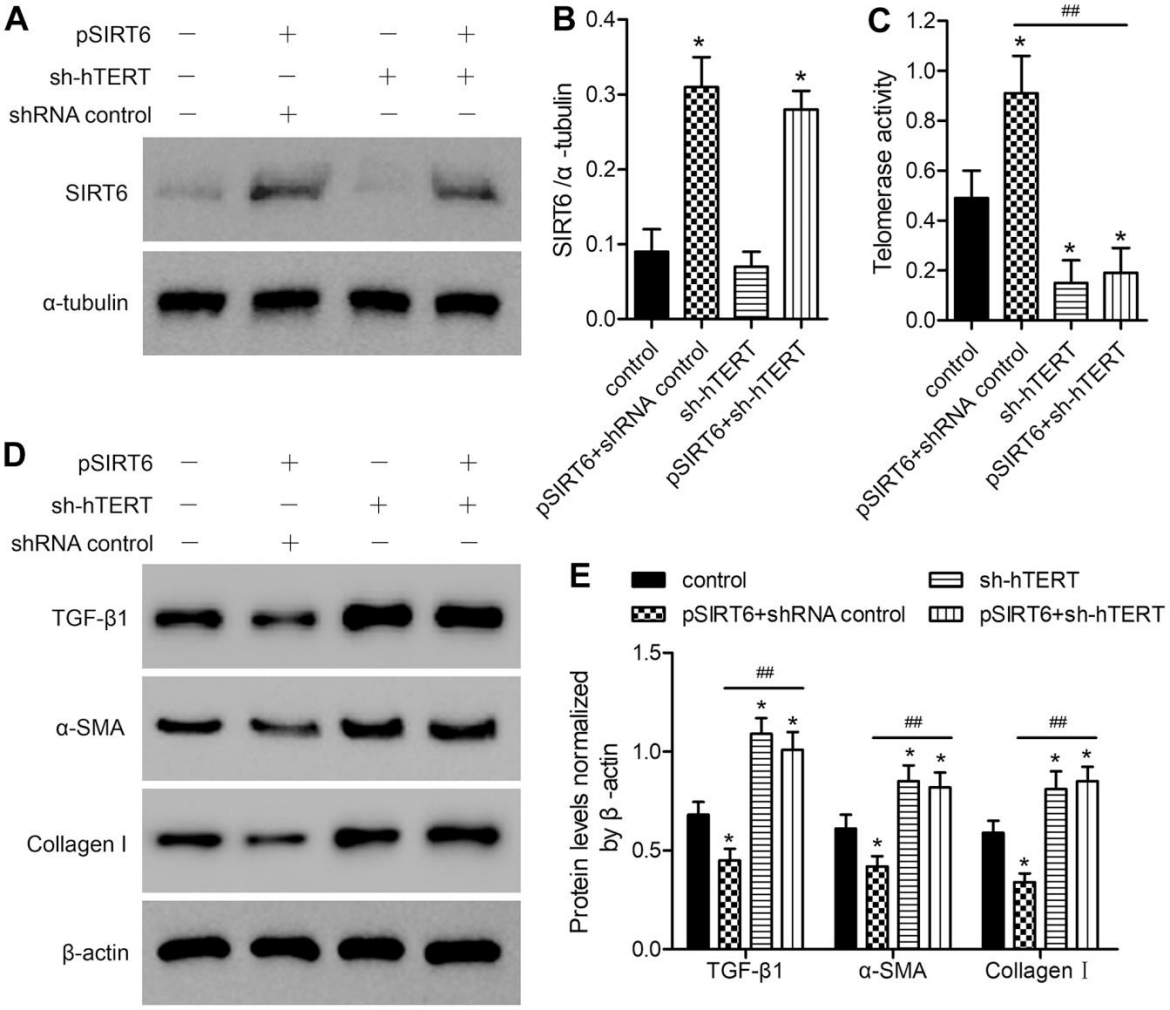
**The impact of SIRT6 overexpression on telomerase activity and fibrosis-related protein expression is reversed by hTERT knockdown.** LFH cells were infected using lentiviral vectors encoding SIRT6 (pSIRT6), an hTERT-specific shRNA (sh-hTERT), or a control shRNA, after which Western blotting was used to assess the expression of SIRT6 in these cells (**A**, **B**), with α-tubulin being used for normalization. (**C**) Telomerase activity in ligamentum flavum cells treated as indicated was assessed based upon optical density. (**D**, **E**) TGF-β1, α-SMA, and collagen I protein levels were analyzed by western blotting, with β-actin being used for normalization. Data are means ± SD of three replicates. *p<0.05, vs. non-infection control. ##*p*<0.01 vs. pSIRT6- and control shRNA-transduced cells (pSIRT6+shRNA control).

To examine the role of telomerase activation in the SIRT6-mediated inhibition of fibrosis in LFH cells, we next utilized an shRNA-encoding lentivirus to knock down hTERT in these same cells. Such knockdown did not adversely impact SIRT6 expression (Figure 4A, 4B and Supplementary Figure 3), but it did reverse SIRT6 overexpression-dependent enhancement of telomerase activity (Figure 4C) and downregulation of TGF-β1, α-SMA, and collagen I (Figure 4D, 4E and Supplementary Figure 4). This, therefore, suggests that SIRT6 can inhibit fibrotic differentiation in LFH cells at least in part via the activation of telomerase activity.

### Knockdown of hTERT reverses the beneficial impact of SIRT6 overexpression on premature cellular senescence in LFH cells

**Figure 5:**
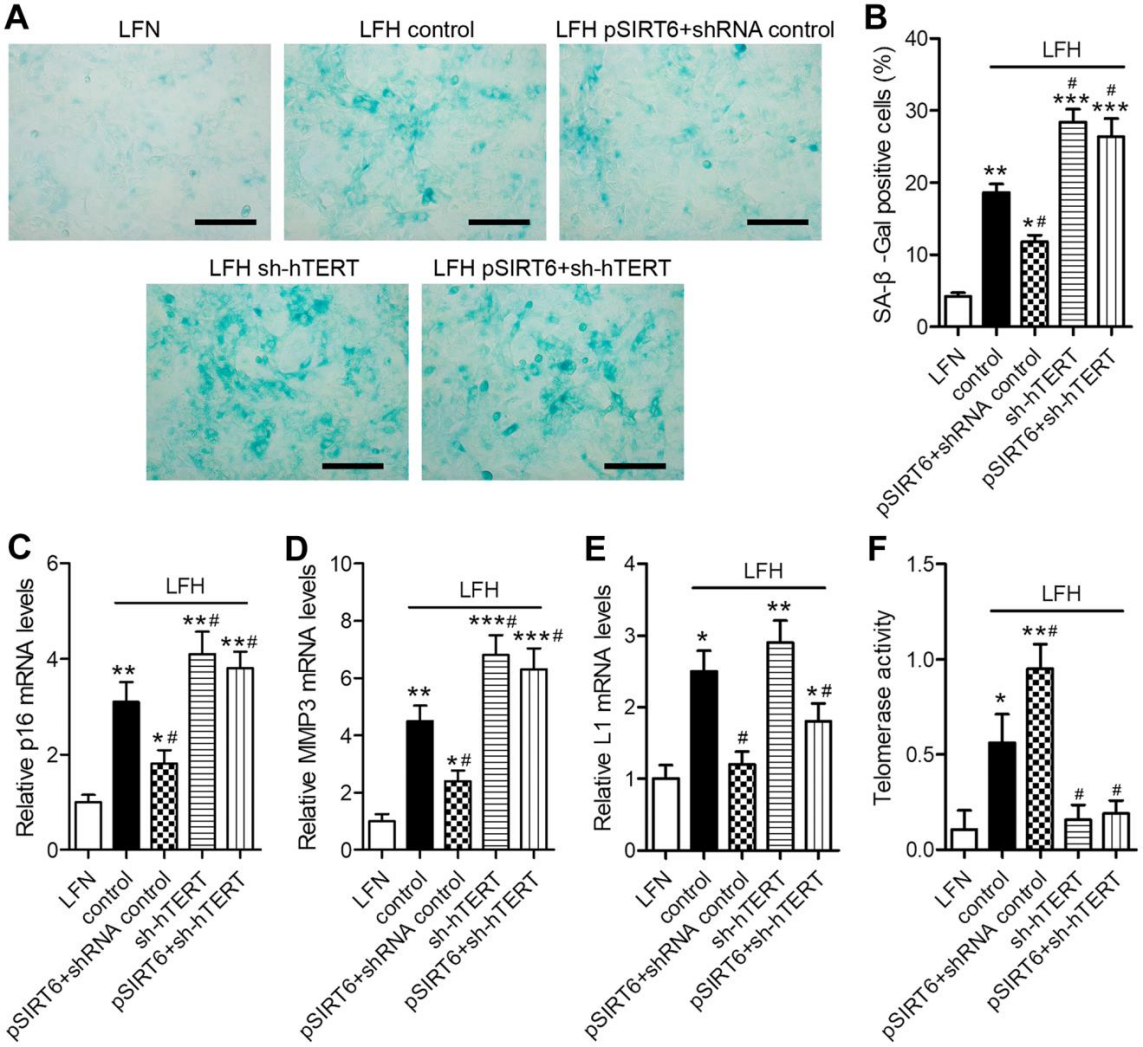
**SIRT6 inhibits LFH cell senescence via promoting telomerase activity.** LFH cells were transduced for 48 h using lentiviruses encoding pSIRT6, sh-hTERT, or a control shRNA, after which senescence-associated β-galactosidase (SA-β-gal) staining of these cells was conducted. Uninfected cells were used as controls. (**A**) Representative bright-field images of SA-β-Gal stained cells. Scale bar = 100 μm. (**B**) SA-β-gal positive cell percentages. mRNA levels of the senescence-related genes p16 (**C**), MMP3 (**D**), and Long interspersed element 1 (L1, **E**) were quantified via qRT-PCR, with GAPDH being used as a normalization control. (**F**) Telomerase activity in ligamentum flavum cells treated as indicated was assessed based upon optical density. Data are means ± SD of three replicates. *p<0.05, **p<0.01, ***p<0.001 vs. LFN. #*p*<0.05 vs. LFH control.

To assess the functional importance of SIRT6 and telomerase activity in the context of LF cellular senescence, we next conducted SA-β-gal staining in these cells at 48 h after SIRT6 overexpression and/or hTERT knockdown (Figure 5A). SA-β-gal staining was increased at baseline in LFH cells relative to LFN cells (18.6% vs. 4.2%). SIRT6 overexpression significantly reduced the frequency of SA-β-gal-positive LFH cells to 11.8%, whereas hTERT knockdown increased this frequency to 28.4%. Importantly, hTERT knockdown in SIRT6-overexpressing cells reversed the beneficial impact of SIRT6 overexpression and increased the frequency of SA-β-Gal positive LFH cells to 26.2% (Figure 5B). Trends in the expression of senescence-related genes including p16^INK4a^, MMP3, and long interspersed nuclear element 1 (L1) were consistent with frequencies of SA-β-gal-positive cells, with the exception of L1, which was inhibited in pSIRT6 and sh-hTERT co-transduced cells, while the same was not true for p16 or MMP3 (Figure 5C–5E). Similarly, hTERT knockdown inhibits telomerase activity regardless of SIRT6 overexpression (Figure 5F). Together, these findings thus suggest that telomerase activity is an essential mediator of the SIRT6-driven inhibition of premature senescence.

### SIRT6 induces telomerase activation and thereby protects LFH cells from DNA damage and telomeric dysfunction

**Figure 6:**
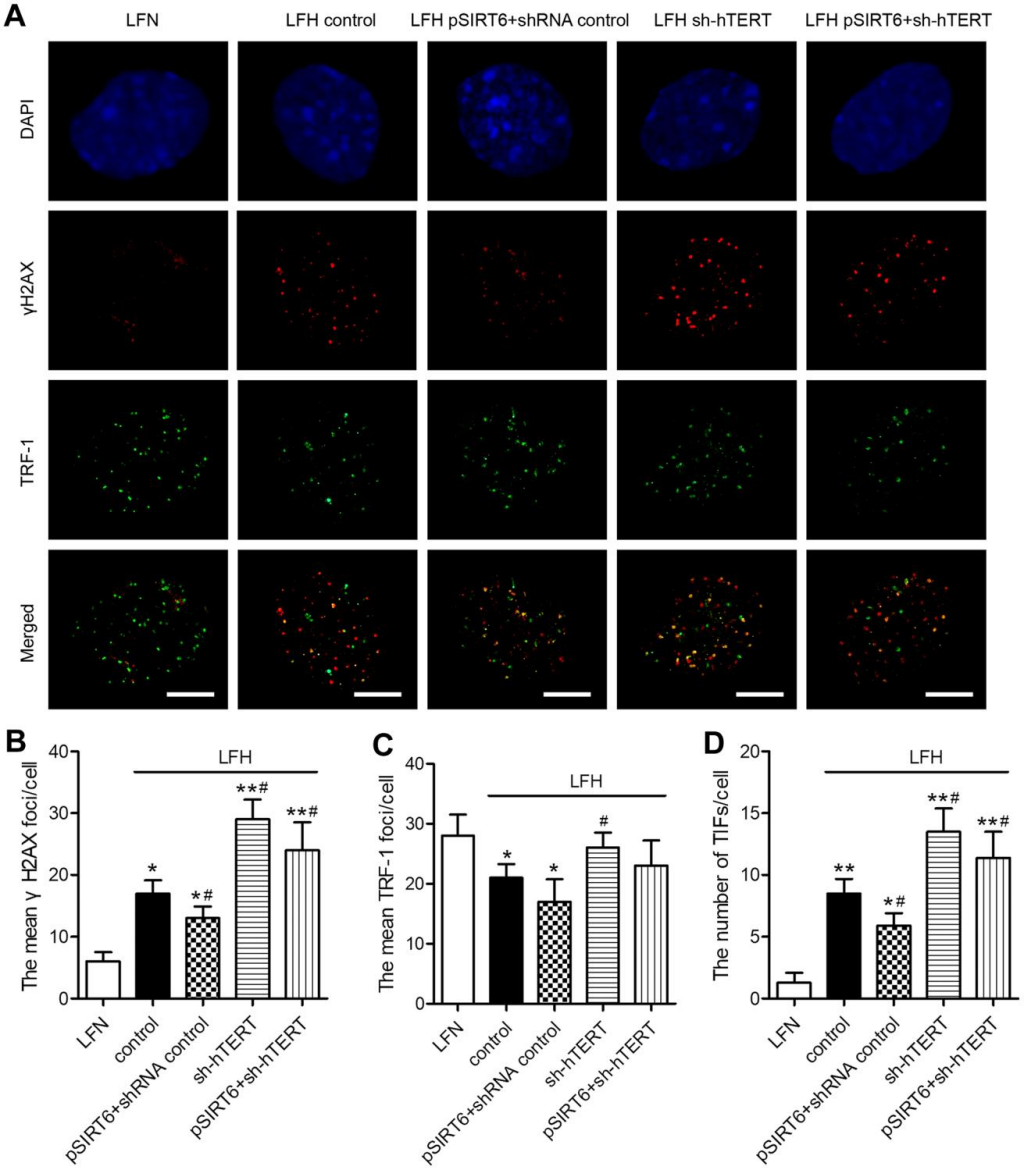
**SIRT6 protects LFH cells from DNA damage and telomere dysfunction via activation of telomerase activity.** (**A**) Representative images of LF cells stained for γH2AX (red) and TRF-1 (green) and counterstained with DAPI (blue). Scale bar = 5 μm. The mean number of γH2AX foci (**B**), TRF-1 (**C**) and telomere dysfunction-induced foci (TIFs, **D**) in cells were quantified using ImageJ. Data are means ± SD of three replicates. *p<0.05, **p<0.01 vs. LFN. #*p*<0.05 vs. LFH control.

To understand the mechanisms whereby SIRT6 overexpression inhibits premature LFH cellular senescence, we next assessed whether such overexpression was associated with any significant changes in γH2AX foci formation or TIF prevalence within these cells, given that these are reliable markers of DNA damage and telomeric dysfunction. We observed significantly higher numbers of γH2AX foci and telomere dysfunction-induced foci (TIFs) in LFH cells, whereas numbers of TRF-1 foci were decreased relative to LFN cells. Importantly, SIRT6 overexpression significantly decreased the numbers of TIFs and γH2AX foci in LFH cells. In contrast, hTERT knockdown was sufficient to significantly increase the number of γH2AX foci and TIFs within LFH cells, thereby reversing the impact of SIRT6 overexpression on the formation of these foci (Figure 6A–6D). Together, these findings suggest that SIRT6 can protect LFH cells against premature senescence via activating telomerase and thereby preventing DNA damage and telomeric dysfunction.

## DISCUSSION

While disc degeneration occurs naturally over the course of human aging, it can be associated with several debilitating conditions including lumbar disc herniation, lumbar canal stenosis, segmental lumbar instability, and degenerative lumbar scoliosis. The hypertrophy of LF cells is a key driver of LSS, and is best characterized by reductions in the relative abundance and organization of elastin fibers within the LF coinciding with increases in the abundance of collagen fibers [3, 4]. Liu et al. [17] previously demonstrated that inhibiting cellular senescence is a potentially viable approach to preventing aging-associated intervertebral disc degeneration and related conditions.

Sirtuins are closely linked to aging responses in mammals, and many studies have demonstrated that SIRT1, SIRT3, and SIRT6 serve as inhibitors of aging-related processes [18–20]. Moreover, SIRT6 acts as a suppressor of aging-related genes such as p16, MMP3, and L1 [21, 22]. In this study, we confirmed that SIRT6 localizes to the nuclei in human LF tissue samples, and we found that it was expressed at much lower levels in LFH cells relative to LFN cells. Importantly, we demonstrated that SIRT6 can prevent both DNA damage and telomeric dysfunction in LF cells, thereby protecting them against premature aging.

The structural and functional integrity of telomeres is dependent upon telomerase-mediated elongation and on the shelterin complex (TRF1, TRF2, TIN2, hRAP1, TPP1, and POT1), which shields these structures from degradation [23]. Telomerase activity is primarily mediated by TERT, which functions as a component of a ribonucleoprotein complex in order to synthesize repeating telomere sequences that are attached to the ends of each chromosome [24]. In a study utilizing TERT-knockout mice, Cheng et al. [25] demonstrated that reduced telomerase activity was associated with increased cellular senescence, reduced autophagy in renal tubular epithelial cells, and impaired renal functional recovery in response to ischemia-reperfusion injury. In a separate analysis, Liu et al. [26] overexpressed hTERT in hepatic tissues from elderly rats prior to liver transplantation and thereby found that hTERT was able to protect these cells from apoptotic death.

Telomerase is present in the nucleus wherein it repairs chromosomal DNA when cells divide or DNA is damaged. As such, telomerase activity is suppressed at baseline in healthy tissues, whereas it is activated in tumors and proliferating cells [27]. Our results clearly demonstrate that LFH tissues exhibited increased TERT and telomerase activity as well as decreased POT1 and TPP1 expression relative to LFN tissues, consistent with a role for enhanced telomerase activity in hyperplasic tissues [27, 28]. Dechsupa et al. [29] demonstrated that LFH cell samples from LSS patients exhibited increased oxidative DNA damage and shorter average telomere lengths relative to LFN cells from these same patients. However, this prior study did not examine telomerase activity or the underlying molecular mechanisms governing this phenotype.

In this study, we found that overexpressing SIRT6 was sufficient to bolster telomerase activity in LFH cells while simultaneously reducing the expression of the fibrosis-related proteins TGF-β1, α-SMA, and collagen I in these same cells. We further confirmed that such SIRT6 overexpression was able to protect LFH cells from telomeric dysfunction and DNA damage. SIRT6 knockdown has previously been demonstrated to drive significant increases in DNA damage and telomeric dysfunction in human chondrocytes [16], whereas SIRT6 overexpression protects against myocardial damage via altering telomeres [30] and prevents myofibroblast differentiation via the consequent inactivation of the TGF-β1/Smad2 pathway [31]. We determined that telomerase inactivation mediated by hTERT knockdown in LFH cells was sufficient to enhance DNA damage, senescence, and myofibroblastic differentiation while also reversing the protective impact of SIRT6 overexpression on these same phenotypes. Razdan et al. [32] previously demonstrated that telomeric dysfunction can drive human fibroblast transdifferentiation into myofibroblasts. Our results are thus consistent with findings from many previous reports, suggesting that SIRT6 overexpression can prevent the myofibroblastic differentiation of LF cells through a mechanism dependent upon changes in the expression and activity of telomerase.

In summary, in the present study we found that SIRT6 overexpression in human LF cells was sufficient to protect them against DNA damage, premature senescence, and myofibroblastic differentiation through a mechanism dependent upon telomerase activation. Future studies regarding the relationship between SIRT6 and telomerase may offer novel insights into the development and progression of LSS, and have the potential to identify important new therapeutic targets for the treatment of this disease.

## MATERIALS AND METHODS

### Patient specimens

LF tissue samples were collected from 33 patients (20 males, 13 females) with LSS at L4/L5 that had undergone decompressive surgery at Renji Hospital (Shanghai, China). Patients were between the ages of 50 and 70 (mean: 58.1±7.4 years). LFH samples were collected from those patients exhibiting evidence of LF hypertrophy at the L4/L5 level, while non-hypertrophic samples from the L3/L4 level in these same patients were collected as LFN controls. Following collection, these LF tissue samples were either snap-frozen and stored at −80° C, or were immediately used to isolate LF cells. The ethics committee of Renji Hospital approved this study, which was consistent with the Declaration of Helsinki. All patients provided written informed consent prior to study participation.

### Sequencing analysis

High-throughput transcriptomic sequencing was used to compare patterns of gene expression in LFH and LFN samples. Briefly, tissue RNA was extracted with TRIzol (Invitrogen, CA, USA), after which chloroform and isopropyl alcohol were used to precipitate this RNA. Sequencing and subsequent bioinformatics analyses were conducted by Cloud-Seq Biotech (Shanghai, China). Briefly, total RNA was treated with a Ribo-Zero rRNA Removal Kits (Illumina) to remove rRNA based on the manufacturer’s instructions, after which RNA libraries were constructed using rRNA-depleted RNAs with a TruSeq Stranded Total RNA Library Prep Kit (Illumina) according to the manufacturer’s instructions. Libraries were then subjected to quality control and quantification using the BioAnalyzer 2100 system (Agilent Technologies, USA), and 10 pM libraries were denatured to yield single-stranded DNA molecules that were captured on Illumina flow cells, amplified *in situ* as clusters and finally sequenced for 150 cycles on an Illumina HiSeq Sequencer as per the manufacturer’s instructions.

### qRT-PCR

TRIzol (Invitrogen) or an RNeasy mini kit (Qiagen, CA, USA) were used to extract RNA from samples, with RNAlater (Qiagen) having been used for RNA preservation. A RetroScript kit (Ambion, TX, USA) was used to reverse transcribe RNA (2 μg/sample) based on provided directions. SYBR Green Rox Master Mix (Qiagen) was used for qRT-PCR reactions, which were performed using an ABI 7300 instrument (Applied Biosystems, CA, USA). GAPDH was used as a normalization control. All primer sequences are compiled in Table 2.

**Table 2:** Primer sequences and production size for qRT-PCR.

**Target gene**	**Upstream sequence**	**Downstream sequence**	**Product (bp)**
TPP1	5'-GAAAGCTGCCTGACACTGGA-3'	5'-TGGGTGAGGAAGGAGGAGAG-3'	253
SIRT6	5'-GTACGTCCGAGACACAGTCG-3'	5'-ATGTACCCAGCGTGATGGAC-3'	206
WRAP53	5'-ACAACCACCTGGATGAGCTG-3'	5'-CGTAGGAGCCACAGGCATAG-3'	225
NOP10	5'-CTCAACGAGCAGGGAGATCG-3'	5'-TGGGTCATGAGCACCTTGAA-3'	155
SMG6	5'-CCAAGAGGGAACACGACTGG-3'	5'-GCCTCATCTGACTGTGGTCC-3'	206
POT1	5'-GAGGACACCACTATGTGCCA-3'	5'-AATCGCCCTCATGTGGAACT-3'	158
p16INK4a	5'-ACTCTCACCCGACCCGTG-3'	5'-AATCGGGGATGTCTGAGGGA-3'	241
MMP3	5'-CCTGGAAATGTTTTGGCCCAT-3'	5'-CATCTTGAGACAGGCGGAAC-3'	235
L1	5'-GTGCACATGTACCCTAAAACTTAGA-3'	5'-GCAAAACGCGGTAAAATGGA-3'	229
GAPDH	5'-TTCACCACCATGGAGAAGGC-3'	5'-GATGGCATGGACTGTGGTCA-3'	240

### LF cell culture

The isolation of LF cells was conducted as in prior studies [33]. Briefly, collected LF tissue was minced into ~0.5-1 mm^3^ cm pieces and was then successively digested with 0.25% trypsin and 250 U/mL type I collagenase (Sigma, USA) in T25 flasks. Cells were then cultured in DMEM containing 10% FBS and 1% penicillin/streptomycin (Gibco, USA) at 37° C in a humidified 5% CO_2_ incubator until confluent. The media was replaced every third day. Trypsin was used to passage cells when confluent, and experiments were conducted using LF cells from the third passage.

### Lentiviral transfection

To overexpress SIRT6 in LF cells, PCR was used to amplify full-length human SIRT6 cDNA from gDNA with the following primers: F, 5’-GCAGTCTTCCAGTGTGGTGT-3’; R, 5’-CACTTCAAGGTGGGTCTCCC-3’. This amplified product was then inserted into the pLVX-IRESpuro vector (System Biosciences, CA, USA). To knock down hTERT, an hTERT-specific short-hairpin RNA (shRNA) (sh-hTERT: 5’-CCATCCGTGTCACCAACTTGT-3’) or a nonspecific control construct (5’-CAGCTCTACATGATCCAGAAA-3’) was inserted into the PLVTHM vector (System Biosciences). HEK293T cells were then transfected with these vectors or control vectors along with appropriate packaging vectors using Lipofectamine 2000 (Invitrogen, CA, USA). At 48 h post-transfection, supernatant samples were collected and were used to infect LF cells for 48 h.

### Western blotting

A mixture of M-PER Tissue Protein Extraction Reagent (Pierce, IL, USA), EDTA, and protease inhibitors (Beyotime, Shanghai, China) was used to extract total protein from LF tissue samples, whereas LF cellular protein was isolated using RIPA lysis buffer containing protease inhibitors (Beyotime). A BCA kit (Beyotime) was then used to quantify protein levels in individual samples. Equal amounts of total protein from each sample (40 μg) were then separated via 10% SDS-PAGE and transferred onto PVDF membranes. Blots were then blocked with 5% SA, followed by overnight incubation with primary antibodies at 4° C. Antibodies were from Abcam (United Kingdom), were prepared in 5% BSA, and were as follows: anti-SIRT6 (#ab62739, 1:2000); anti-TGF-β1(#ab215715, 1:1000); anti-α-SMA (#ab7817, 1:3000); anti-Collagen I (#ab260043, 1:5000); anti-α-tubulin (#ab7291, 1:1000); and anti-β-actin (#ab8226, 1:5000). Following an additional 1 h incubation with HRP-conjugated secondary antibodies (#ab6789, 1:2000; #ab205718, 1:50000), protein bands were detected with an enhanced chemiluminescence kit (Millipore, Germany). The ImageJ software was then used for densitometric quantification, with α-tubulin or β-actin being used for normalization purposes.

### Immunohistochemistry (IHC)

Paraffin-embedded LF tissue samples were cut into 4μm-thick sections, after which they were deparaffinized using xylene. Sections were then treated using 3% H_2_O_2_ in order to quench endogenous peroxidase activity, followed by a 30-minute incubation with citrate buffer in a steamer to mediate antigen retrieval. Sections were then stained overnight with anti-TPP1 (#ab54685, 1:100, Abcam), anti-POT1 (#10581-1-AP, 1:200, Proteintech, Wuhan, China), anti-SIRT6 (#ab62739, 1:200, Abcam), or anti-TERT (#382962, 1:200, ZenBio, Chengdu, China) at 4° C, followed by a 45-minute incubation with HRP-linked goat anti-rabbit IgG (#ab205718, 1:20000, Abcam) or goat anti-mouse IgG (#ab6789, 1:500, Abcam) at room temperature. DAB was then used for specific protein detection in these samples, which were then rinsed and counterstained with hematoxylin for 30 seconds. Samples were then analyzed via light microscopy (Olympus Optical, Tokyo, Japan) and assessed with the ImageJ software.

### Telomerase activity analysis

Extracts from LF tissues or cells were used to conduct the telomeric repeat amplification protocol (TRAP reaction). Telomerase activity was quantified using the Telo TAGGG Telomerase PCR ELISA kit (Roche, Mannheim, Germany) according to the manufacturer’s instructions.

### Senescence associated β-galactosidase (SA-β-Gal) assay

SA-β-Gal staining was conducted as in prior reports by using the chromogenic 5-bromo-4-chloro-3-indoyl-β-D-galactopyranoside substrate [29, 34]. Briefly, appropriately treated LF cells were added to 6-well plates (1×10^5^/well) for 48 h at 37^°^ C. Cells were then washed with PBS, fixed with 4% paraformaldehyde (PFA) for 15 minutes at room temperature, washed with PBS, and stained with an SA-β-gal staining solution (40 mM citric acid/sodium phosphate [pH 6.0], 150 mM NaCl, 2 mM mgCl_2_, 5 mM potassium ferrocyanide, 5 mM potassium ferricyanide, and 1 mg/mL of 5-bromo-4-chloro-3-indoyl-β-D-galactopyranoside) for 16 h at 37° C. Light microscopy was then used to quantify the relative frequency of SA-β-gal-positive LF cells [29]. Overall counts were the mean of three separate counts per sample.

### Immunofluorescent staining

DNA damage and telomere dysfunction were quantified as in previous studies via the detection of γH2AX foci corresponding to phosphorylated histone H2AX and telomere dysfunction-induced foci (TIFs) corresponding to regions wherein γH2AX foci co-localize with telomere repeat binding factor-1 (TRF-1) [35]. Briefly, cells that had been plated onto coverslips were first permeabilized for 10 minutes using 0.25% Triton X-100, after which they were fixed for 15 minutes using 4% PFA, re-permeabilized with Triton X-100, and blocked using 1% BSA (Sigma). Samples were then probed for 1 h with anti-TRF-1 (#ab10579, 1:500, Abcam) and anti-γH2AX (#9718, 1:200, Cell Signaling), followed by incubation with appropriate AF488- and AF594-conjugated IgGs, respectively (#A-11029, 1:500; # A-21207, 1:1000; Invitrogen). Samples were then washed with PBST, nuclei were counterstained using DAPI, and the ProLong Gold Antifade reagent (Invitrogen) was used to mount samples prior to their analysis via immunofluorescence confocal microscopy (Nikon Inc., Tokyo, Japan). ImageJ was used to quantify the average number of γH2AX foci, TRF-1 foci and TIFs (γH2AX foci co-localized with TRF-1 foci) per cell.

### Statistical analysis

Data are means ± standard deviations (SDs) from three independent experiments. GraphPad Prism 5 (GraphPad, CA, USA) was used to compare data via unpaired Student’s t-tests or one-way ANOVAs with Tukey’s post hoc test as appropriate. A two-tailed P < 0.05 was considered to be statistically significant.

## Supplementary Material

Supplementary Figures

Supplementary Table 1
