# Supplementary material for: SIRT6 enhances telomerase activity to protect against DNA damage and senescence in hypertrophic ligamentum flavum cells from lumbar spinal stenosis patients
Source: Aging (Albany NY). 2021 Feb 10;13(4):6025–40. doi: 10.18632/aging.202536 (PMC7950242; doi:10.18632/aging.202536)
Supplement: Supplementary Figures [file aging-13-202536-s001.pdf]

SUPPLEMENTARY FIGURES

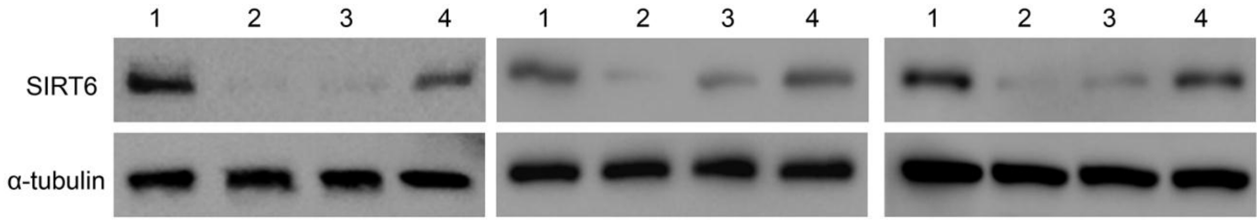

Supplementary Figure 1. SIRT6 or control lentiviral vectors were used to transduce LFH cells for 48 h, after which western blotting was used to assess SIRT6 levels in these cells. α-tubulin was used for normalization. Uninfected LFH cells and LFN cells were included as controls. 1, LFN; 2, LFH-control; 3, LFH-vector; 4, LFH-pSIRT6.

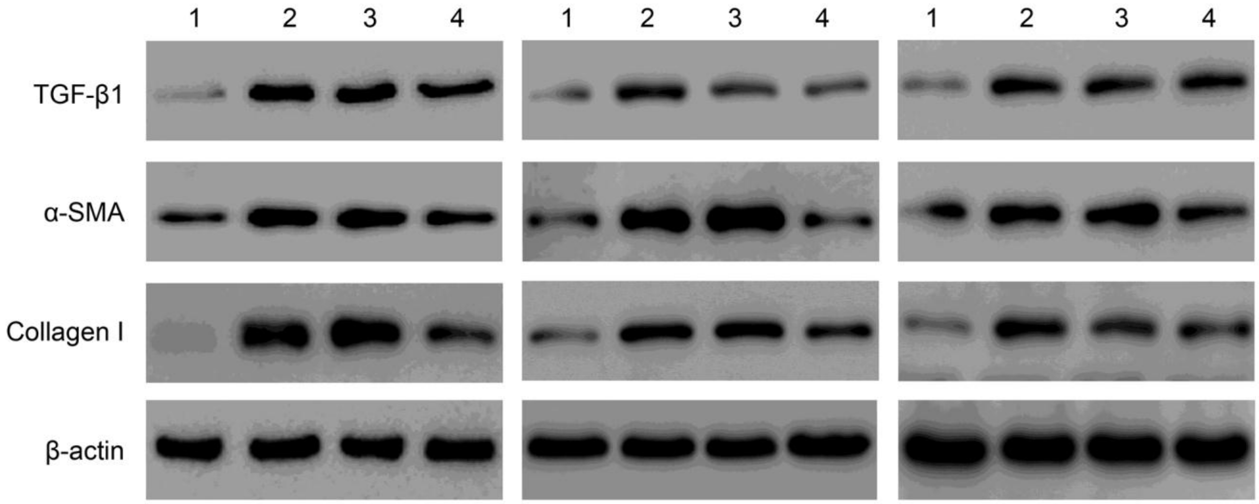

Supplementary Figure 2. SIRT6 or control lentiviral vectors were used to transduce LFH cells for 48 h, TGF-β1, α-SMA, and collagen I protein levels were analyzed by western blotting. β-actin was used as a loading control. Uninfected LFH cells and LFN cells were included as controls. 1, LFN; 2, LFH-control; 3, LFH-vector; 4, LFH-pSIRT6.

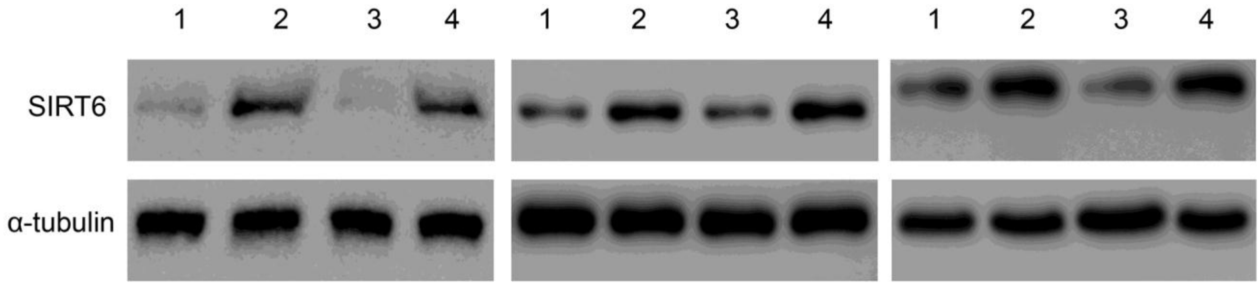

Supplementary Figure 3. LFH cells were infected using lentiviral vectors encoding SIRT6 (pSIRT6), an hTERT-specific shRNA (sh-hTERT), or a control shRNA, after which Western blotting was used to assess the expression of SIRT6 in these cells, with α-tubulin being used for normalization. 1, non-infection control; 2, pSIRT6 + shRNA control; 3, sh-hTERT; 4, pSIRT6 + sh-hTERT.

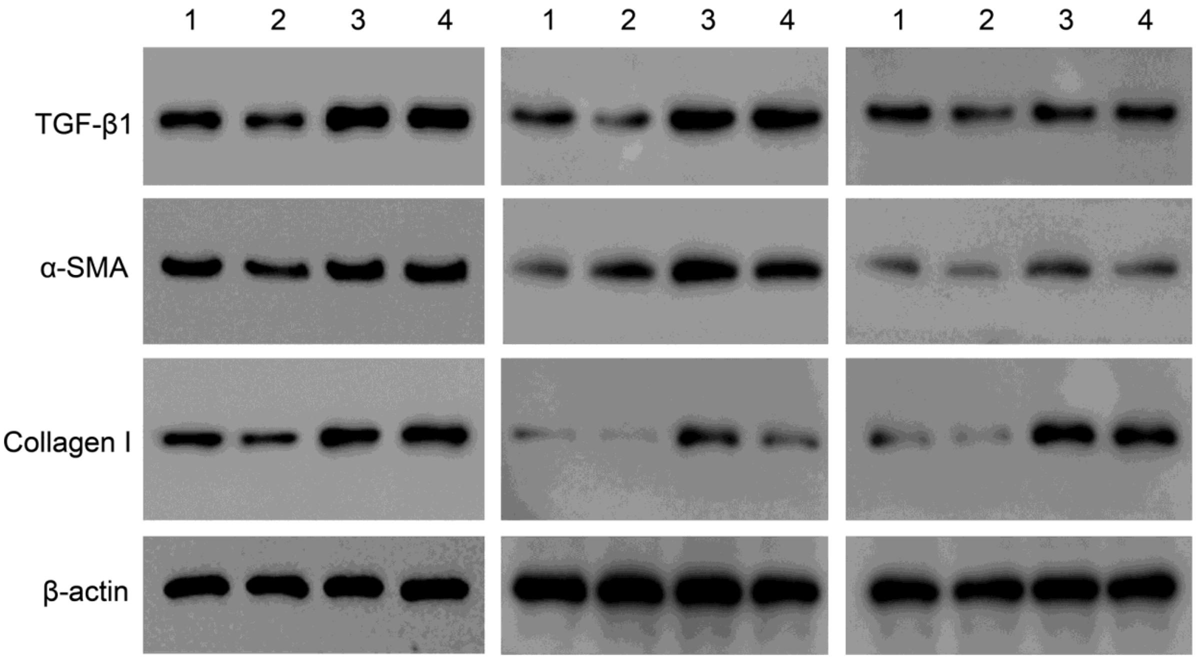

Supplementary Figure 4. LFH cells were infected using lentiviral vectors encoding SIRT6 (pSIRT6), an hTERT-specific shRNA (sh-hTERT), or a control shRNA, after which, TGF-β1, α-SMA, and collagen I protein levels were analyzed by western blotting, with β-actin being used for normalization. 1, non-infection control; 2, pSIRT6 + shRNA control; 3, sh-hTERT; 4, pSIRT6 + sh-hTERT.
